# Supplementary material for: Mmu-let-7a-5p inhibits macrophage apoptosis by targeting CASP3 to increase bacterial load and facilities mycobacterium survival
Source: PLoS One. 2024 Sep 3;19(9):e0308095. doi: 10.1371/journal.pone.0308095 (PMC11371246; doi:10.1371/journal.pone.0308095)
Supplement: S1 Raw images — (PDF) [file pone.0308095.s001.pdf]

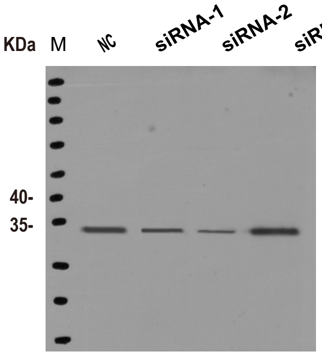

Fig 3.D Caspase3

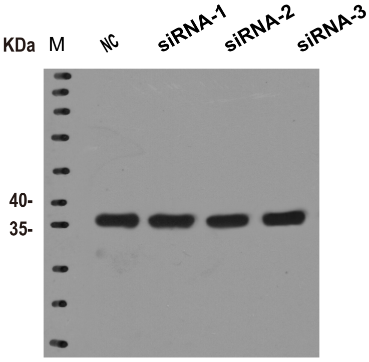

Fig 3.D GAPDH

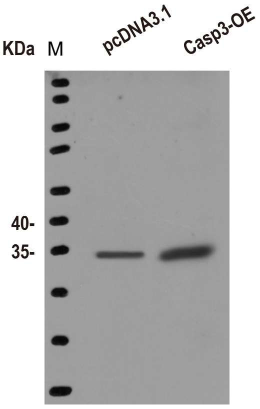

Fig 3.E Caspase3

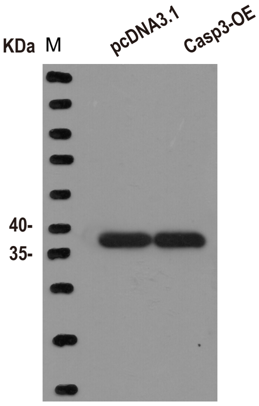

Fig 3.E GAPDH

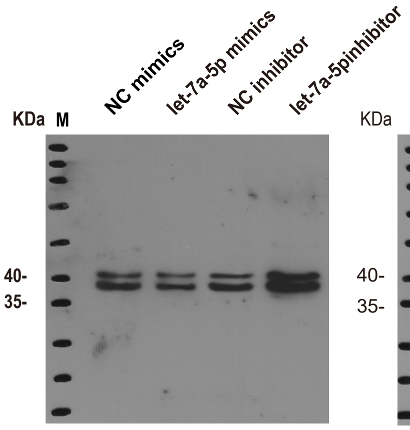

Fig 5.B Caspase-3

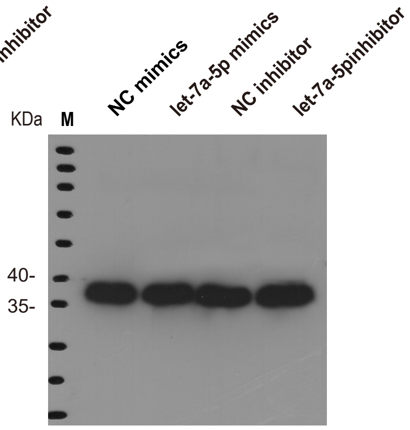

Fig 5.B GAPDH

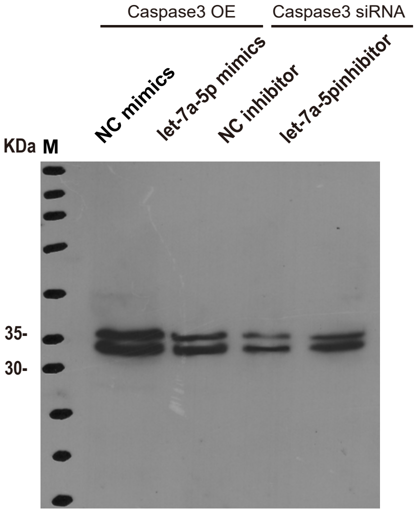

Fig 5. D Caspase-3

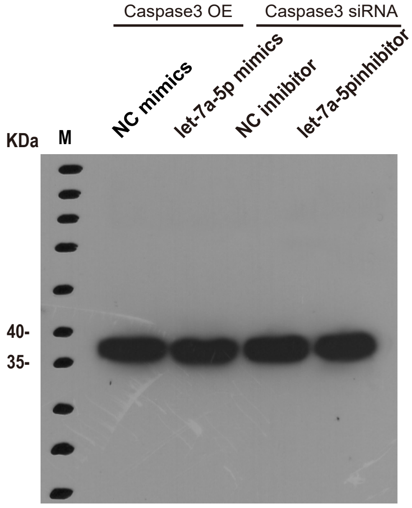

Fig 5.D GAPDH

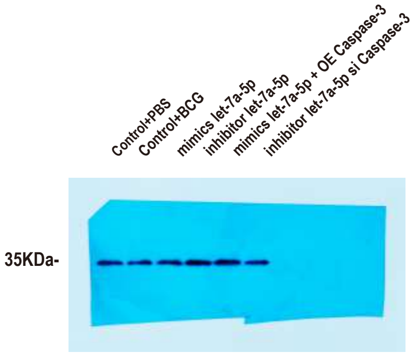

Fig 8.E Caspase-3

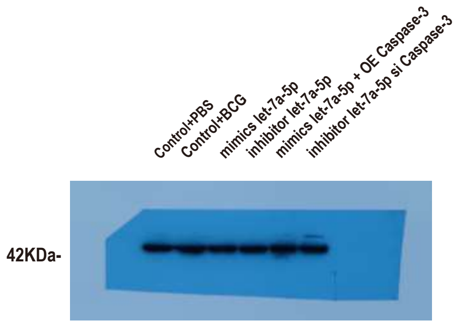

Fig 8.E β-actin

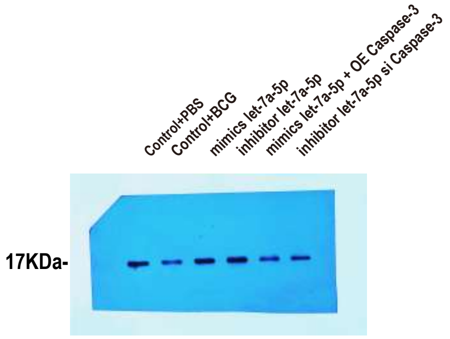

Fig 8.E Caspase-3 cleaved

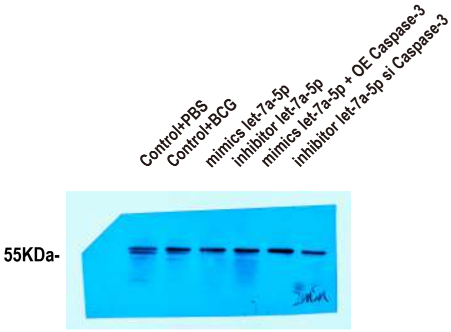

Fig 8.E TNFR1

Description: protein quantification 54ug, Antibody 1: 1000 dilution, hypersensitive exposure 10s.
