# Supplementary material for: Mmu-let-7a-5p inhibits macrophage apoptosis by targeting CASP3 to increase bacterial load and facilities mycobacterium survival
Source: PLoS One. 2024 Sep 3;19(9):e0308095. doi: 10.1371/journal.pone.0308095 (PMC11371246; doi:10.1371/journal.pone.0308095)
Supplement: Attachment — Submitted filename: review comment1.docx [file pone.0308095.s002.docx]

Review comments

This study found that mmu-let-7a-5p regulates the mycobacterial load in macrophages through apoptosis, which is of certain value and innovation, because in addition, reports about it focus on tumors and cardiovascular diseases, and few reports are related to infectious diseases. But there are some issues in the article that need to be revised

1. The figure legends were not clear, and there was no explanation of the production method or number of replicates for each group of samples.

2. There are A-H graphs in Figure 8, but only A-B is explained and does not match

3. There are some columnar statistical charts, although the p-value is less than 0.05, the actual difference is not significant, which usually means that there may be some deviation between the statistical significance and the actual biological significance. As shown in Figure 8F, Figure 7B, etc

4. Figure 8G and Figure 8H, qRT PCR to detect mRNA of Caspase-3 Pro and Caspase-3 cleared. In fact, qRT PCR cannot distinguish between Caspase-3 Pro and Caspase-3 cleared. You need to explain.
