# Supplementary material for: Mmu-let-7a-5p inhibits macrophage apoptosis by targeting CASP3 to increase bacterial load and facilities mycobacterium survival
Source: PLoS One. 2024 Sep 3;19(9):e0308095. doi: 10.1371/journal.pone.0308095 (PMC11371246; doi:10.1371/journal.pone.0308095)
Supplement: Attachment — Submitted filename: Response to Reviewers'.docx [file pone.0308095.s003.docx]

**Response to Reviewers**

Dear Editors and Reviewers:

Thank you for your letter and for the reviewer’s comments concerning our manuscript entitled “**Mmu-let-7a-5p inhibits macrophage apoptosis by targeting CASP3 to increase bacterial load and promote facilities mycobacteria survival**” [manuscript number: PONE-D-23-31553].

First of all, please let me express my respect and sincere thanks for your conscientious work attitude. Those comments are all valuable and very helpful for revising and improving our manuscript, as well as the important guiding significance to our researchers.

***Review comments***

*This study found that mmu-let-7a-5p regulates the mycobacterial load in macrophages through apoptosis, which is of certain value and innovation, because in addition, reports about it focus on tumors and cardiovascular diseases, and few reports are related to infectious diseases. But there are some issues in the article that need to be revised*

*1. The figure legends were not clear, and there was no explanation of the production method or number of replicates for each group of samples.*

*2. There are A-H graphs in Figure 8, but only A-B is explained and does not match*

*3. There are some columnar statistical charts, although the p-value is less than 0.05, the actual difference is not significant, which usually means that there may be some deviation between the statistical significance and the actual biological significance. As shown in Figure 8F, Figure 7B, etc*

*4. Figure 8G and Figure 8H, qRT PCR to detect mRNA of Caspase-3 Pro and Caspase-3 cleared. In fact, qRT PCR cannot distinguish between Caspase-3 Pro and Caspase-3 cleared. You need to explain.*

***Reply***

We have studied comments carefully and have made correction which we hope meet with approval. Revised portion are marked in red in the paper. The main corrections in the paper and the responds to the reviewer’s comments are as flowing:

1. *The figure legends were not clear, and there was no explanation of the production method or number of replicates for each group of samples*.

Thank you for your comments. We have revised our figure legends and annotated description of replicates for each group of samples.

2. *There are A-H graphs in Figure 8, but only A-B is explained and does not match*

We are very sorry for our negligence of these mistakes in the manuscript writing and we have carefully revised these mistakes.

3. *There are some columnar statistical charts, although the p-value is less than 0.05, the actual difference is not significant, which usually means that there may be some deviation between the statistical significance and the actual biological significance. As shown in Figure 8F, Figure 7B, etc*.

We are extremely grateful to Reviewer for pointing out this problem. We have double checked the raw data and re-calculated the statistics by using Graphpad Prism 9.0. There are indeed significant differences in the results of statistical charts in our manuscripts.

4. *Figure 8G and Figure 8H, qRT PCR to detect mRNA of Caspase-3 Pro and Caspase-3 cleared. In fact, qRT PCR cannot distinguish between Caspase-3 Pro and Caspase-3 cleared. You need to explain.*

Due to our negligence, we did not explain exactly about the figure legends, especially Figure 8. In fact, Figure 8.F-H were the quantitative analysis of TNFR1, Caspase-3 Pro and Caspase-3 cleaved protein expression in Western Blotting, they were not the expression level of mRNA.

***Additional Editor Comments***

*This study aims to explore the impact of mmu-let-7a-5p on Raw264.7 mononuclear macrophage apoptosis and its influence on intracellular Bacillus Calmette-Gu&eacute;rin (BCG) load in vitro. While the study holds potential interest for readers, there are several significant changes required.*

*1. The abstract section needs enhancement, specifically in introducing the theme, outlining the aims, and summarizing methods and results. Additionally, the conclusion section should outline future research directions, as the current presentation is unclear.*

*2. The introduction section should also be improved with background information relating to the study&#39;s aims. Furthermore, the material and methods section would benefit from the inclusion of a schematic diagram illustrating the study&#39;s protocol and a justification for using GAPDH as a reference gene for qPCR detection of mRNA. Additionally, the authors should provide the original figures of the western blot as supplementary files for the results section and submit the main raw data as supplementary files.*

*3. In the discussion section, the authors should refrain from comparing the present study&#39;s data to their previous findings and expand the analysis to include international data. Moreover, it should be important to insert the section limitations.*

*4. Lastly,* *the conclusion section should suggest future research directions.*

***Reply***

1. *The abstract section needs enhancement, specifically in introducing the theme, outlining the aims, and summarizing methods and results. Additionally, the conclusion section should outline future research directions, as the current presentation is unclear*.

Thank you for your comments. Considering the Editor’s suggestion, we have re-written the abstract section part.

Abstract: We have been trying to find a miRNA that can specifically regulate the function of mycobacterial host cells to achieve the purpose of eliminating Mycobacterium tuberculosis. The purpose of this study is to investigate the regulation of mmu-let-7a-5p on macrophages apoptosis and its effect on intracellular BCG clearance. After a series of in vitro experiments, we found that mmu-let-7a-5p could negatively regulate the apoptosis of macrophages. by targeting Caspase-3. The extrinsic apoptosis signal axis TNFR1/FADD/Caspase-8/Caspase-3 was inhibited after BCG infection. Up-regulated the expression level of mmu-let-7a-5p increase the cell proliferation viability and inhibit apoptosis rate of macrophages, but down-regulated its level could apparently reduce the bacterial load of intracellular Mycobacteria and accelerate the clearance of residual Mycobacteria effectively. Mmu-let-7a-5p has great potential to be utilized as an optimal candidate exosomal loaded miRNA for anti-tuberculosis immunotherapy in our subsequent research.

2. *The introduction section should also be improved with background information relating to the study&#39;s aims. Furthermore, the material and methods section would benefit from the inclusion of a schematic diagram illustrating the study&#39;s protocol and a justification for* *using GAPDH as a reference gene for qPCR detection of mRNA.* *Additionally, the authors should provide the original figures of the western blot as supplementary files for the results section and submit the main raw data as supplementary files*.

Thank you for your comments and we have updated the background information of the introduction section. Furthermore, a schematic diagram was supplied to the material and methods.

Glyceraldehyde-3-phosphate Dehydrogenase (GAPDH) is widely present in many organisms and is abundant in cells, accounting for 10% -20% of the total protein. The gene has a highly conserved sequence, which is highly expressed in almost all tissues. The protein expression in the same cell or tissue is generally constant, and it is not affected by the induction substances such as partial recognition check point and phorbol lipid. It has been commonly used as a reference gene in RT-qPCR assays for gene and protein expression analysis. Due to this, we chose GAPDH as a reference gene for qPCR detection of mRNA.

Additionally, we have re-submitted the original figures of the western blot as supplementary files for the results section and submit the main raw data as supplementary files.

3. *In* *the discussion section, the authors should refrain from comparing the present study&#39;s data to their previous findings and expand the analysis to include international data. Moreover, it should be important to insert the section limitations*.

Thank you for your comments. We have modified the discussion section according to your comments.

4. *The conclusion section should suggest future research directions*.

Thank you for your comments. Based on our primary research, we will expect to construct a new multiple drug delivery system with exosomes for tuberculosis treatment in the future, which loaded miRNA and traditional anti-tuberculosis drug rifampin. This was mentioned in the conculsion.

Conclusion: Mmu-let-7a-5p negatively regulates the apoptosis of Raw264.7 macrophages by targeting Caspase-3. Down-regulated the expression of mmu-let-7a-5p facilities the clearance of residual mycobacteria and attenuation the bacterial load of intracellular mycobacteria. Therefore, mmu-let-7a-5p has the great potential to be utilized as a candidate miRNA for the construction of novel exosomal drug delivery system loading with rifampin for anti-tuberculosis therapy in our subsequent research.

***Minor points:***

*-* *English should be reviewed by a native speaker.*

*-* *Please, check the use of reference manager software (Error! Reference source not found..-&gt; line 121).*

*-* *The short form should be introduced by the extension form the first time; subsequently, it should be used through the text. Please, check all abbreviations.*

***Reply***

*1. English should be reviewed by a native speaker.*

Thank you for your advice. We not only invited an English-speaking friend from the United States to help polish our manuscript. Our manuscripts are also edited by one or more highly qualified native English editors of AJE for appropriate English language, grammar, punctuation, spelling and overall style.

*2. Please, check the use of reference manager software (Error! Reference source not found.-&gt; line 121).*

Thank you for your comments. We are very sorry for our negligence of the mistake in line 121 of the manuscript and we have revised the mistake.

*3. The short form should be introduced by the extension form the first time; subsequently, it should be used through the text. Please, check all abbreviations.*

According to the Reviewer’s suggestion, we have double checked all the abbreviations in our manuscript.

We tried our best to improve the quality of our manuscript and made some changes to the manuscript. These changes will not influence the content and framework of the paper. And we not only listed the changes in the email but also marked in red in the revised manuscript. We appreciate Editors / Reviewers’ warm work earnestly, and hope that the correction will meet with approval.

Once again, thank you very much for your comments and suggestions.

Yours sincerely

Xuehua Zhan

Corresponding author: Zhaohui Ge

E-mail: myovid@126.com
