# Supplementary material for: Mmu-let-7a-5p inhibits macrophage apoptosis by targeting CASP3 to increase bacterial load and facilities mycobacterium survival
Source: PLoS One. 2024 Sep 3;19(9):e0308095. doi: 10.1371/journal.pone.0308095 (PMC11371246; doi:10.1371/journal.pone.0308095)
Supplement: Attachment — Submitted filename: Response to Reviewers.docx [file pone.0308095.s004.docx]

**Response to Reviewers**

Dear Editors and Reviewers:

Thank you for your response and for the reviewer’s comments concerning our manuscript entitled “**Mmu-let-7a-5p inhibits macrophage apoptosis by targeting CASP3 to increase bacterial load and promote facilities mycobacteria survival**” [manuscript number: PONE-D-23-31553R1].

Please let me express my respect and sincere thanks for your conscientious work attitude. Those comments are all valuable and very helpful for revising and improving our manuscript, as well as the important guiding significance to our researchers.

***Journal Requirements***

*Please review your reference list to ensure that it is complete and correct. If you have cited papers that have been retracted, please include the rationale for doing so in the manuscript text, or remove these references and replace them with relevant current references. Any changes to the reference list should be mentioned in the rebuttal letter that accompanies your revised manuscript. If you need to cite a retracted article, indicate the article’s retracted status in the References list and also include a citation and full reference for the retraction notice.*

**Reply**

We have reviewed our references and double-checked the references list, no article was retracted.

***Review comments***

*Reviewer #3: The re-submitted manuscript entitled “Mmu-let-7a-5p inhibits macrophage apoptosis by targeting CASP3 to increase bacterial load and facilities mycobacterium survival” by Zhan et al have made considerable modifications, however, there are still some minor concerns that need further addressed before accept.*

*1.* *Line 253 “3.7 Mmu-let-7a-5p enhances Raw264.7 cell proliferation and viability”, according to Fig 6 and its-related contents, the current subtitle of 3.7 needs to rethink and refine, especially only CCK-8 results whether could support the cell proliferation as authors claim?*

*2.* *Figure 7, if possible, suggests the authors add a group that is just Raw264.7 cells without anything as a negative control. The current apoptotic data from Nc-mimics and NC-siRNA almost reached 30%, whether the Nc-mimics and NC-siRNA could promote apoptosis? To exclude the possibility of higher apoptotic rates such as Nc-mimics and NC-siRNA groups, the cell picture from each should provide additional support.*

*3.* *In Fig 10, it’s better to add the dilution index on the top of panel A.*

*4**. Considering the large amounts of data and results of this study, a graphic abstract is helpful if it is provided.*

***Reply***

We have studied comments carefully and have made correction which we hope meet with approval. Revised portion are marked in red in the paper. The main corrections in the paper and the responds to the reviewer’s comments are as flowing:

1. *Line 253 “3.7 Mmu-let-7a-5p enhances Raw264.7 cell proliferation and viability”, according to Fig 6 and its-related contents, the current subtitle of 3.7 needs to rethink and refine, especially only CCK-8 results whether could support the cell proliferation as authors claim?*

Thank you for your comments and we are extremely grateful to Reviewer for pointing out this problem. Considering the reviewer’s comment, we have revised our subtitle of 3.7 and figure legends of Fig 6. The CCK-8 results are appropriate to demonstrate the cell viability.

2. *Figure 7, if possible, suggests the authors add a group that is just Raw264.7 cells without anything as a negative control. The current apoptotic data from Nc-mimics and NC-siRNA almost reached 30%, whether the Nc-mimics and NC-siRNA could promote apoptosis? To exclude the possibility of higher apoptotic rates such as Nc-mimics and NC-siRNA groups, the cell picture from each should provide additional support.*

First of all, we thank you for your rigorous and realistic academic style. In our data, there is a blank cell group which is Raw264.7 cells without anything as a negative control as you referred to above. Because the processing of apoptosis data needs to be compared with blank cells, the histogram shown later is actually the value compared with blank cells. Although the apoptosis rate of the Nc-mimics group and the NC-siRNA group was nearly 30%, they were significantly different from the statistical analysis results between the let-7a-5p mimics group and the Casp-3 siRNA group.

3. *In Fig 10, it’s better to add the dilution index on the top of panel A.*

Thank you for your comments. Considering the reviewer’s suggestion, we have added the dilution index on the top of panel A.

4. *Considering the large amounts of data and results of this study, a graphic abstract is helpful if it is provided.*

Thank you so much for your affirmation and suggestions for our work. In fact, we have already prepared a graphic abstract before our first submission. We will upload the graphic abstract together this time. Please review and make clear whether it is suitable for publication.

Finally, we sincerely thank the reviewers for their valuable comments and suggestions. We try our best to revise the manuscript and improve the quality of our manuscripts by making some modifications. These changes will not affect the content and framework of the paper. We sincerely thank the editors / reviewers for their enthusiastic work and hope that the review work will be recognized.

Yours sincerely

Zhaohui Ge
